# The Development of Computational Biology in Pakistan: Still a Long Way to Go

**DOI:** 10.1371/journal.pcbi.1001135

**Published:** 2011-06-30

**Authors:** Muhammad Ilyas, Samrene Sadique, Kahlid Masood, Raheel Qamar, Shahid Nadeem Chohan

**Affiliations:** 1 Bioinformatics Laboratory, National Centre of Excellence in Molecular Biology, University of the Punjab, Lahore, Pakistan; 2 Department of Biosciences, COMSATS Institute of Information Technology, Islamabad, Pakistan; 3 Shifa College of Medicine, Islamabad, Pakistan; 4 Centre for Plants and the Environment, University of Western Sydney, NSW, Australia; University of California San Diego, United States of America

**Author profiles**

**Muhammad Ilyas** is a PhD Research Scholar at the National Centre of Excellence in Molecular Biology (CEMB), University of the Punjab, Lahore, and has worked as Visiting Research Student at the Korean Bioinformation Center (KOBIC), South Korea. He is the founder president of the International Society for Computational Biology Regional Students Group of Pakistan (ISCB-RSG-Pakistan) (milyaskh@hotmail.com ).

**Samrene Sadique** is currently working as a research officer in the Bioinformatics Laboratory at the CEMB, University of the Punjab, Lahore (sas1069@londonmet.ac.uk ).

**Khalid Masood** is Principal Investigator of Bioinformatics Laboratory at the CEMB, University of the Punjab, Lahore (khalid@cemb.edu.pk ).

**Raheel Qamar** is the Dean of the Faculty of Sciences at the COMSATS Institute of Information Technology (CIIT) and manager of the Pakistan EMBnet National Node. He has worked as a resource person for several bioinformatics workshops in Pakistan and played a pioneering role in introducing Bioinformatics in the country (raheel_qamar@comsats.edu.pk ).

**Shahid Nadeem Chohan** is a member of ISCB and faculty advisor for Pakistan ISCB-RSG. He is also founder of the Pakistan EMBnet National Node at CIIT and elected member of the Education and Training Project Committee of the EMBnet. He has worked as a resource person for several bioinformatics workshops in Pakistan. He is currently working as a Higher Education Commission (HEC) foreign faculty at CIIT, Islamabad, (snchohan@hotmail.com).

Different researchers from across the globe are currently using computer technology coupled with biological research to answer biologically relevant questions. Computational biology is playing a fundamentally important role in the development of society by providing information quickly and facilitating the research process. This field of study was introduced to Pakistan in 2003, when undergraduate degree programs in Bioinformatics were created in two different universities. Such institutions have now increased to 14. Since the introduction of the field in Pakistan many workshops and conferences have also been organized by public and private institutions. A few enthusiastic students of bioinformatics in the country have also started a regional group, ISCB-RSG-Pakistan (http://www.iscbsc.org/rsg/rsg-pakistan) as well as hosting a bulletin board (http://groups.google.com/group/ISCB-RSG-Pakistan) where students and researchers communicate with each other, discuss their projects, share their knowledge, and enhance their exposure in the field of study. The purpose of this article is to review the current situation of computational biology and bioinformatics research, as well as the availability of resources and the awareness among students in Pakistan. In our opinion, these are important parameters which will play a fundamental role in improving bioinformatics and computational biology research in Pakistan.

## Introduction

Scientific research has played a major role in the betterment of humanity. Due to ongoing research in the area of biological sciences a huge amount of valuable data has been generated in recent years and it is estimated that these data are almost doubling each year [1]. This immense amount of data requires ample storage, easy updating, and accessibility to all the researchers around the world [2].

Computers and the Internet have become an integral part of research in different areas of science and technology; without these tools rapid advancement is no longer possible [3]. Use of these tools for addressing problems in molecular biology has given rise to new disciplines termed computational biology or bioinformatics [4]. These disciplines are relatively new fields in Pakistan where they were recently introduced, first by various workshops and then by the introduction of different undergraduate degree programs.

## The Rise of Bioinformatics in Pakistan

Pakistan is rich in genetic resources, such as its diverse human population, crops, and other species. These resources have proved useful in understanding and solving a range of biological problems particularly relevant to this part of the world. In agriculture, for example, numerous new varieties of a number of field crops have been developed, most notably of cereals [1]. However, in some other important and interesting fields of research, such as drug development, protein expression assays, population genetics, and clinical trials, much room for further development remains. Such progress may be achieved through more effective utilization of the skilled human resources available in Pakistan, and result in significant advancement in scientific research [1].

**Figure 1:**
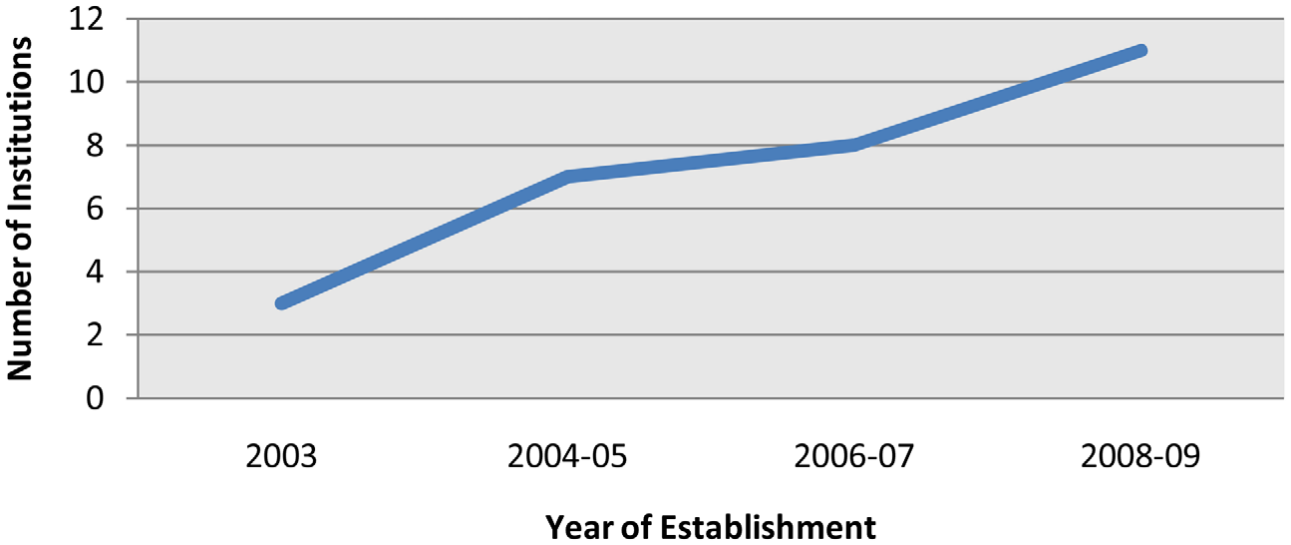
The growth of bioinformatics research and degree-awarding institutes in Pakistan.

Bioinformatics was introduced in Pakistan in 2003, when two undergraduate degree programs in this field were introduced in Islamabad at the COMSATS Institute of Information Technology (CIIT) and Muhammad Ali Jinnah University. These two institutions are considered pioneers in the field of bioinformatics education in Pakistan. Later, similar bachelor degree programs in this discipline were introduced in other universities, such as the International Islamic University (Islamabad), Government College University (Faisalabad), and the University of Veterinary and Animal Sciences (Lahore). The number of such programs has progressively grown to about 11 different institutions offering an undergraduate degree program in the field (Figure 1), while a few universities have also launched postgraduate degrees (MS and PhD) in bioinformatics and related fields (Table 1).

**Table 1:** Bioinformatics research and degree awarding public and private institutes in Pakistan.

No.	Name of Institution	Website	Program
1.	COMSATS Institute of Information Technology, Islamabad	http://www.ciit.edu.pk/	BS/MS
2.	Muhammad Ali Jinnah University, Islamabad	http://www.jinnah.edu.pk/	BS/MS/PhD.
3.	International Islamic University, Islamabad	http://www.iiu.edu.pk/	BS/MS
4.	Government College University, Faisalabad	http://www.gcuf.edu.pk/	BS
5.	Bahria University, Karachi	http://www.bimcs.edu.pk/	Research
6.	Centre of Excellence in Molecular Biology, Lahore	http://www.cemb.edu.pk/	Research
7.	Panjwani Center for Molecular Medicine and Drug Research, Karachi	http://www.iccs.edu/	Research
8.	Quaid-e-Azam University, Islamabad	http://www.qau.edu.pk/	MS/MPhil
9.	Institute of Molecular Sciences & Bioinformatics, Lahore	http://www.imsb.edu.pk/	Research
10.	Balochistan University of Information Technology, Engineering and Management Sciences, Quetta	http://www.buitms.edu.pk/	BS
11	University of Veterinary and Animal Sciences, Lahore	http://www.uvas.edu.pk	BS
12	Al-Khawarizmi Institute of Computer Science (KICS), Lahore	http://www.kics.edu.pk	Research
13	Lahore University of Management Sciences, Lahore	http://www.lums.edu.pk	Research
14	Baqai Institute of Information Technology, Karachi	http://www.baqai.edu.pk	BS

## Including Bioinformatics in the Biology Curriculum

Because of the importance of bioinformatics to the biological sciences, in 2006 the Higher Education Commission of Pakistan (HEC) defined a curriculum for a BS bioinformatics degree program and advised all public and private universities to include bioinformatics as a subject in the curriculum of all undergraduate biology degree programs(http://www.hec.gov.pk/InsideHEC/Divisions/AECA/CurriculumRevision/Documents/Bioinformatics-2006.pdf).

This step could play a major role in popularizing the area of research amongst potential future researchers of Pakistan in the field of biology. To date only a small number of institutions have adopted this advice; the rest of them, hopefully, will follow in the near future.

## Careers for Bioinformatics Graduates

Graduates of most professional fields have the option of joining the corresponding industry in Pakistan. However, this option is not available to local bioinformatics graduates, because the bioinformatics industry simply does not yet exist. It has been estimated through personal observations and interviews that roughly 250 bioinformatics graduates are being produced in the country every year with a significant gender bias tilting towards females. No published data are available but it has also been observed that many female graduates leave study and take up family responsibilities as they get married. The remaining graduates overall continue to study at the postgraduate level in bioinformatics, locally or internationally. Sweden is the most popular destination due to the high quality of education available and the absence of any tuition fees for international students. Some of the graduates leave the field of bioinformatics and join biosciences based on wet laboratory research. The remaining minority transfers either to computer sciences or entirely different areas such as business administration. Most of the local or returning postgraduates find placements within the bioinformatics and biosciences faculties in the local institutions in the country.

## Human Resource Development in Pakistan

Training a new skilled workforce and developing the existing one play a significant role in developing a novel discipline in any country. For this purpose local graduates need to be trained at the postgraduate level (MS / PhD) in other countries where this field is more established and advanced.

Realizing this fact, the Pakistan EMBnet National Node at the CIIT lobbied the HEC to allocate funds for this purpose. Following successful lobbying the HEC, realizing the importance of this field of study, approved 50 PhD scholarships in bioinformatics, and the first 16 scholars were sent to Sweden. This was done with the intention that once these and subsequent scholars returned from their studies they would be inducted in groups of five in ten different current and future units working in the area of bioinformatics. It was hoped that this would result in a significant boost in research and teaching in Bioinformatics in Pakistan. Since its inception, the HEC has funded human resource development in the higher education area of the country through a sustained funding initiative providing scholarships for study and research in developed countries as well as locally. Information about such programs is available on their website (http://www.hec.gov.pk/InsideHEC/Divisions/HRD/Scholarships/ForeignScholarships/90OverseasScholarshipsP2B2/Pages/Default.aspx).

In addition to various teaching activities since 2003, a few workshops and conferences have also been supported financially by the HEC on bioinformatics in highly ranked institutions such as the Dr. Panjwani Center for Molecular Medicine and Drug Research (Karachi), CEMB, University of the Punjab (Lahore), CIIT/COMSTECH Secretariat (Islamabad), and Kohat University of Science and Technology (Kohat). Some self-supported mini-workshops were also arranged by a number of universities (Table 2).

**Table 2:** Bioinformatics workshop and conferences organized in Pakistan.

No.	Title	Organizer	Date
**1**	International Workshop on Bioinformatics	COMSTECH and PTCL Pakistan	22–25 April 2003
**2**	Virtual Conference on Bioinformatics and Genomics	Dr. Panjwani Center for Molecular Medicine and Drug Research Karachi	21–24 September 2004
**3**	Workshop on Bioinformatics	Dr. Panjwani Center for Molecular Medicine and Drug Research Karachi	4 October 2004
**4**	First Computational Chemistry Workshop	Dr. Panjwani Center for Molecular Medicine and Drug Research Karachi	26–28 June 2006
**5**	HEC sponsored Training Course for Researchers to Equip with Latest Bioinformatics Tools	Kohat University of Science and Technology, Kohat (NWFP), Pakistan	15–17 April 2005
**6**	Pre – 18TH FAOBMB Symposium Satellite Workshop on Bioinformatics	Center of Excellence in Molecular Biology, Lahore	14–19 November 2005
**7**	4th International Symposium on Genetic Engineering & Biotechnology, titled "Genetics, Bioinformatics, Biotechnology and Economic Development"	Centre for Molecular Genetics, Genetics Department, Karachi University, Karachi, Pakistan	4–8 December 2005
**8**	International Thematic Workshop on the "Use of Bioinformatics in Genomics Research”	COMSTECH and COMSATS Institute of Information Technology	19 August-2 September 2006
**9**	National Workshop on Bioinformatics for Computer Scientists	COMSTECH and COMSATS Institute of Information Technology	21–25 May 2007
**10**	A workshop on Bioinformatics	Centre for Molecular Genetics, at L.E.J. National Science Information Center, University of Karachi, Karachi, Pakistan	28–30 June 2007
**11**	1st National Workshop for Computational Biology & Bioinformatics	Bioinformatics Research Lab, Bahria University, Karachi	3–5 April 2009
**12**	Bioinformatics: Opening up new frontiers in molecular biology research	Institute of Biochemistry & Biotechnology University of Veterinary and Animal Sciences, Lahore	14–15 May 2010

## Joining International Forums in Bioinformatics

The International Society for Computational Biology (ISCB), a global organization dedicated to the advancement of scientific understanding of living systems through computation, provides an excellent forum for the interaction of researchers in the area of bioinformatics, which is highly beneficial for the members. However, only a handful of researchers from Pakistan have joined this society. Although there are special rates for the subscription fee, it is still expensive by local standards. Moreover funding for participation in the events organized by the ISCB is very limited.

A significant step was taken in 2006 when CIIT was elected as EMBnet Pakistan National Node. This step provided international exposure for local bioinformaticians and opportunities to collaborate with the international players in the field. It was through this collaboration that the University of Uppsala (Sweden) designed a special postgraduate program for Pakistani graduates who were sponsored by HEC. The students were selected by an international panel comprising Erik Bongcam-Rudloff, Shahid Nadeem Chohan, Raheel Qamar, and a representative from HEC. The EMBnet also helped set up a local server, called e-Biokit, which offers an online suite of bioinformatics programs to local users and is currently available only within CIIT [5].

## Promoting Liaisons with the Local Industry

In order to promote bioinformatics in Pakistan, liaisons between researchers and the local industry must be promoted as well. The International Islamic University took a step in this direction by organizing a national seminar in 2010, “Bioinformatics and Pakistani Industries seminar”(http://www.thefreelibrary.com/InternationalIslamicUniversityseminaronBioinformatics,Pakistan…-a0241676116).

The seminar attracted an audience from across Pakistan and was attended by representatives from universities, the HEC, and industry as represented by the President of the Chamber of Commerce. More seminars like this would stimulate interaction between academia and industry, which will be very useful for the overall development of computational biology in Pakistan. This will require concerted efforts by both the industry and the academic and research communities of the country.

In the absence of local bioinformatics industry in the country, the academic and research institutions can also come forward with initiatives to launch new companies based on the spin-off company's model. Such an initiative is currently underway by the CIIT. A concept paper has been developed and submitted to the CIIT to set up a new company that will employ about 30 fresh bioinformatics graduates. These graduates will have on-the-job experience while carrying out commercially viable projects under the supervision of experienced faculty who will be on the board of directors of this company. The concept paper was prepared by three faculty members of the CIIT (Raheel Qamar, Shahid Chohan, and Nazim Rahman).

## Bioinformatics Scientific Societies

A professional national society can provide a very useful platform for productive professional interaction among local scientists and researchers. Keeping this in mind, the Bio-informatics Society of Pakistan was announced during a conference at the School of Biological Sciences, Punjab University in 2008. This news was covered by the local media (http://www.daily.pk/bio-informatics-society-established-at-punjab-university-pakistan-4756/). The mission of this society was to promote the exchange of ideas and the development of infrastructure and resources in the fields of bioinformatics and computational biology, and to facilitate interaction and collaboration among scientists and educators around the country. Unfortunately, since the conference no further progress in this area has been achieved, and nothing substantial has been done by the society since its formation.

However, due to the personal interest and enthusiasm of bioinformatics students from different universities of Pakistan, a regional student group (RSG-Pakistan) was established in 2010. This group, which is affiliated with the ISCB, was intended as a platform for students to communicate with each other, discuss their projects, and share their knowledge. The primary goal of RSG-Pakistan is to help students gain exposure in the field of computational biology; the group has also set up a bulletin board where students discuss their problems and research activities (http://groups.google.com/group/ISCB-RSG-Pakistan).

The RSG-Pakistan is providing a virtual forum for the mutual interaction and benefit of the local bioinformatics students, researchers, academicians, and industry representatives. Currently there are coordinators representing seven universities and coordinators from local industry are now being sought. As there is no bioinformatics industry in Pakistan, the local computer industry may be the best candidate for this purpose. Moreover, national and local bioinformatics related events are also advertised on their website (http://www.iscbsc.org/rsg/rsg-pakistan). The RSG-Pakistan is also trying to organize virtual internships, virtual conferences, seminars, and collaborations with other domestic societies and groups in order to organize and expand its national base and further activities in the field. Although this forum does not provide direct support to students for admissions or job searches, it has the potential to facilitate their suitable placement in research institutions or industry.

## Current Bioinformatics Research in Pakistan

Recently several tertiary educational institutions in Pakistan have started offering postgraduate programs in bioinformatics and, with them, research groups are also being established in these institutions. Some of these groups are conducting basic research, while a few of them are making impressive progress in novel areas. Three examples of the increasing number of programs in Pakistan follow; their research papers can be found in a number of peer-reviewed journals or in online indexes such as PubMed.

- The Bioinformatics Research Group (BRG) was established at the CIIT, Islamabad (http://cub.comsats.edu.pk). This group is working on comparative plant (rice and *Arabidopsis*) genomics, plant promoter prediction, and poly (A) site prediction in eukaryotes. The work done in this research group is funded by the HEC and has been published internationally. In addition, freely accessible software developed by BRG is available on their web server. This research group was set up by Dr. Ilham Shahmuradov, a scientist from Azerbaijan who worked in Pakistan as HEC foreign faculty for three years at CIIT, Islamabad.
- The Computational Chemistry Group is currently working in the Dr. Panjwani Center for Molecular Medicine and Drug Research, the University of Karachi. This group is headed by Dr. Zaheer-ul-Haq Qasmi, a researcher who obtained postdoctoral training in the area of drug design in Austria. The research in this group involves the application and development of all aspects of medicinal chemistry, organic synthesis, molecular modeling, computational chemistry, computer-aided drug design, virtual screening (docking, scoring, 3D-QSAR; CoMFA, COMSIA), virtual combinatorial library design using pharmacophore approaches, protein structure prediction, and molecular dynamics simulations. This research is funded by several grants from various agencies such as HEC, the Pakistan Science Foundation, and the British Council. The research is also published in international journals (http://www.iccs.edu/hej/faculty2.php; http://sites.google.com/site/zaheerqasmi/ ).
- A few years ago, an institute was set up in Lahore by Dr. Nasir ud Din, an expatriate Pakistani scientist returning from the United States. The Institute of Molecular Sciences and Bioinformatics provides facilities for research in *in silico* biology. Their research aims include (http://imsb.edu.pk/home.htm) mechanisms of multifunctional behavior of proteins, transitory proteins, transcription factors, transitory genes, and modification of proteins and glycoproteins; modification potential of proteins relevant to phosphorylation and glycosylation on hydroxy and amino functions of amino acids; and, in future programs prediction of modification potential of proteins as it relates to the multifunctional nature of proteins in vivo.

## Suggestions for Further Development of Bioinformatics in Pakistan

A few suggestions that would help in creating awareness among the scientific community of Pakistan and encourage prospective scientists and students are listed below:

- Bioinformatics courses should be included in the curriculum of all degree qualifications in the area of biology. While this will introduce the area to the students it will also create more jobs within the country for bioinformatics graduates, consequently making this field more attractive to prospective undergraduates.
- In addition to creating a new skilled workforce by offering undergraduate degree programs in bioinformatics, existing graduates in biology, computer science, statistics, mathematics, etc. should be offered postgraduate diploma programs to enrich the skill sets of working scientists. This will produce more tangible results in a shorter period of time.
- Cross-discipline seminars can be very helpful in attracting prospective graduate students from allied disciplines like computer science, statistics, and mathematics.
- As major biological research in Pakistan is mostly undertaken in universities, these institutes should arrange regular annual seminars, conferences and workshops in order to encourage interaction among experts, academicians, researchers and students etc. In this way students will become acquainted with the latest advancements in bioinformatics and will design their thesis research projects accordingly.
- Universities like Allama Iqbal Open University and the Virtual University of Pakistan should arrange distance learning diplomas and short courses for professionals who want to develop basic knowledge of bioinformatics.
- A major step towards improving bioinformatics research in Pakistan would be projects conducted in collaboration with foreign universities and research institutes.
- At present, there is not a single national journal of bioinformatics. The creation of such a journal would promote local research in the field.
- Articles written by bioinformatics experts and published in the local print and electronic media can play an important role in spreading knowledge of computational biology amongst students and the general public.
- Seminars and career counseling events organized to introduce bioinformatics to students at the senior school level as an alternative career could result in the attraction of more prospective graduates to bioinformatics.
- The bioinformatics curriculum taught at all universities should be accredited in order to set the standard of bioinformatics courses and to ensure that these standards are met accordingly. This can be done by the HEC.
- More bioinformatics resources can be developed locally and provided online to local and international users. As a first step, local mirror sites for popular online resources may be established and maintained. This step will immensely increase the local expertise in developing bioinformatics resources.
